# New Insights on the Nickel State Deposited by Hydrazine Wet-Chemical Synthesis Route in the Ni/BCY15 Proton-Conducting SOFC Anode

**DOI:** 10.3390/nano11123224

**Published:** 2021-11-27

**Authors:** Dimitrinka Nikolova, Margarita Gabrovska, Gergana Raikova, Emiliya Mladenova, Daria Vladikova, Krassimir L. Kostov, Yordanka Karakirova

**Affiliations:** 1 Institute of Catalysis, Bulgarian Academy of Sciences, 1113 Sofia, Bulgaria; margarita.gabrovska@abv.bg (M.G.); daniepr@ic.bas.bg (Y.K.); 2 Acad. Evgeni Budevski Institute of Electrochemistry and Energy Systems, Bulgarian Academy of Sciences, 1113 Sofia, Bulgaria; graikova@iees.bas.bg (G.R.); e_mladenova@iees.bas.bg (E.M.); d.vladikova@iees.bas.bg (D.V.); 3 Institute of General and Inorganic Chemistry, Bulgarian Academy of Sciences, 1113 Sofia, Bulgaria; klkostov@svr.igic.bas.bg

**Keywords:** Ni/BCY15 proton-conducting anode, hydrazine reduction, Ni–Ce interaction, SOFC, electrochemical impedance spectroscopy, XPS, EPR

## Abstract

Yttrium-doped barium cerate (BCY15) was used as an anode ceramic matrix for synthesis of the Ni-based cermet anode with application in proton-conducting solid oxide fuel cells (pSOFC). The hydrazine wet-chemical synthesis was developed as an alternative low-cost energy-efficient route that promotes ‘in situ’ introduction of metallic Ni particles in the BCY15 matrix. The focus of this study is a detailed comparative characterization of the nickel state in the Ni/BCY15 cermets obtained in two types of medium, aqueous and anhydrous ethylene glycol environment, performed by a combination of XRD, N_2_ physisorption, SEM, EPR, XPS, and electrochemical impedance spectroscopy. Obtained results on the effect of the working medium show that ethylene glycol ensures active Ni cermet preparation with well-dispersed nanoscale metal Ni particles and provides a strong interaction between hydrazine-originating metallic Ni and cerium from the BCY15 matrix. The metallic Ni phase in the pSOFC anode is more stable during reoxidation compared to the Ni cermet prepared by the commercial mechanical mixing procedure. These factors contribute toward improvement of the anode’s electrochemical performance in pSOFC, enhanced stability, and a lower degradation rate during operation.

## 1. Introduction

In July 2020, the European Commission launched the European Clean Hydrogen Strategy alongside the Strategy for Energy System Integration. Investment in hydrogen will be a critical growth engine in the context of recovery from the COVID-19 crisis and in the longer term, an important component of Europe’s industrial competitiveness.

The production and energy-related consumption of hydrogen by 2050–2100 is expected to exceed the current level by tens or even hundreds of times [1]; accordingly, its multiple utilization is expected to grow and evolve.

The fuel cell industry uses hydrogen as feedstock, which is considered the most environmentally friendly fuel. Nowadays, hydrogen and fuel cell technologies offer greater personal choice in the transition to a low-carbon economy. Hydrogen fuel cells have become a key part since they are seen as reliable emission-free generating systems, alternative to polluting processes based on conventional processes of fossil fuel combustion. Hydrogen and fuel cells are seeing resurgence in interest: large-scale production of fuel cell vehicles has begun, and hundreds of thousands of homes are now heated and powered by fuel cells [2]. Thus, the development of hydrogen fuel cells, producing clean electricity by electrochemical reaction, has an important role in the improvement of human living conditions.

Solid oxide fuel cells (SOFCs) are a promising technology that can provide efficient and clean energy production, generating power from hydrogen, natural gas, and other renewable fuel. They have a number of advantages, such as flexibility towards the type of fuel, ability to tolerate the presence of impurities, higher efficiency, and application of non-noble metal catalysts. For their commercialization, however, lower cost and better durability of the performance are needed. The main pathway to realize these objectives is the reduction of the operating temperature. The conventional SOFCs operate at very high temperatures within 800–1000 °C [3,4,5,6,7,8].

In the moment, the commercial goal is 600–700 °C. These conditions can increase cell stability, improve materials’ compatibility, and ensure cheaper metallic alloys. Since the limitations of the temperature decrease come mainly from the reduced performance of the active materials, approaches for increasing the electrocatalytic activity and ionic conductivity are highly appreciable. The lower activation energy of proton-conducting oxide materials has made them attractive candidates for electrolyte materials operating in the intermediate temperature range (500–700 °C), which opened the direction of the proton-conducting solid oxide fuel cells’ (pSOFCs) development [4].

The principal disadvantage of classical SOFC and of its proton-conducting modification (pSOFC) is the formation of water at the electrodes, where it mixes with the fuel/oxidizer, resulting in electromotive force losses and decreased catalytic activity and durability. A successful attempt has been reported to eliminate water production at the electrodes with the so-called dual-membrane fuel cell (dmFC) design. The latter introduces a separate compartment (central membrane, CM) for the production and evacuation of water. It has mixed ionic (proton and oxide ion) conductivity, which ensures penetration of the protons and oxide ions produced at the electrode/electrolyte interface. They react in the CM to form water that is evacuated through the porous microstructure. This original approach opens a new niche for the development of reversible flexible fuel cells operating at 600–700 °C due to the application of proton-conducting oxide materials [9,10,11,12,13,14].

The type of the electrolyte has a large impact on the optimum cell performance through its contribution to the ohmic internal resistance. For many years, barium cerate-based materials have been known as the electrolyte material with high performance in terms of proton conductivity [4], in particular the yttrium-doped barium cerate, BaCe_0.85_Y_0.15_O_2.925_ (BCY15). Since BCY15 exhibits a high level of oxide ion and proton conduction at medium temperatures (600–700 °C), the central membrane in the dmFC has been simplified by using a single material with sufficient mixed ionic conductivity as BCY15 [14,15,16,17,18].

In responding to the needs of modern society for the replacement of critical components in electrocatalysts from platinum group metals [19], nickel continues to be a non-precious metal of particular interest for the development of the cermet anodes, not only for its catalytic properties, but also for the effective cost reduction for the production of inexpensive, environmentally friendly energy and storage systems.

By analogy with classical cermet anodes, a composite Ni/BCY15 anode has been applied for dmFC construction, and thus the difference in the thermal expansion coefficient between the electrolyte and anode was avoided. Generally, Ni-based cermet production presents incorporation of commercial NiO powder in BCY electrolytes by the traditional oxide powder mixture reaction, consisting of mixing, cold pressing, and sintering at high temperatures (1100–1400 °C) [20,21]. During the solid-state reaction, cationic Ni^2+^→Ce^4+^ partial substitution occurs in the BCY structure [22,23].

Reduction treatment of the anode before the operation of the cell under H_2_/Ar atmosphere at high temperatures (700–800 °C) is then performed to produce metallic Ni, which leads to a morphology change of the composite, bringing about an increase of the anode porosity [22,24,25,26].

Our investigations on electrolyte-supported half-cell BCY15-NiO/BCY15/BCY15-NiO with cermet deposition by tape casting and sintering at temperatures below 1250 °C, which ensure the required porosity, showed a drastic increase of both the resistivity of the electrolyte and the area-specific resistance (ASR) of the anode. TEM/STEM analysis registered an extensive precipitation of nanosized Y_2_O_3_ particles along the NiO/BCY15 and the anode/BCY15 electrolyte interface. Their concentration decreases towards the depth of the electrolyte. Thus, an efficient electrical barrier between the protonic (BCY15) and the metallic (Ni) phase is produced. This phenomenon may be initiated by slight diffusion of Ba toward NiO. For sintering temperatures above 1350 °C, an improvement of both the ASR and resistivity of the electrolyte was observed. X-ray diffraction analysis shows that the system is stabilized towards segregation of yttria, however, the density is under the required limits and a small quantity of a new cerium yttrium oxide phase is registered [26,27].

To overcome the foregoing problems of the commercial mechanical mixing procedure, a new and original approach to metallic Ni introduction in the anode ceramic matrix of BCY15 is introduced based on wet-chemical synthesis of Ni/BCY15 cermet. It is cost-effective and energy-efficient, offering better structural control over the Ni metal particles in the BCY15 matrix for the production of anodes with good connectivity in both metallic and electrolyte phases, satisfactory porosity for the gas transport, and high electrical conductivity. In addition, it is expected that the sintering procedure based on the newly developed Ni/BCY cermet, which will induce partial or full oxidation of the Ni particles and possible ejection out of the Ni-YSZ/air surface, will lead to relief of the internal stress and thus to increased durability [28], which also needs to be checked.

Our expectations were to reach higher Ni^0^ dispersion based on the investigations of Wojcieszak et al. The authors have found that the hydrazine reduction method is better to prepare a Ni/CeO_2_ catalyst with higher dispersion and smaller particle size compared to the conventional H_2_ reduction [29]. The synthesis procedure is based on concomitant nickel introduction in the anode ceramic matrix and gaining metallic Ni particles on the electrolyte surface via wet-reduction by hydrazine reducing agent [30]. The formation of finely divided metal particles proceeds in alkaline solution [31,32,33] and can be summarized by the reaction: 2Ni^2+^+N_2_H_4_+4OH^−^→2Ni^0^+N_2_+4H_2_O. Fundamentally, the severe problem regarding the ceramic BCY structure was the low chemical stability of barium cerate to water [34]. Our challenge was to protect the BCY15 matrix during hydrazine wet-reduction by application of non-aqueous medium [35]. We used ethylene glycol as a protective agent and solvent, but also because of its reduction properties. Ethylene glycol is known as a reducing agent used to obtain nanocrystalline metallic powders by employing the polyol method [36].

This work is focused on the detailed comparison between Ni/BCY15 cermets obtained by hydrazine wet-reduction using two types of media: aqueous medium and anhydrous ethylene glycol environment. The target of this evaluation is to explain the impact of the applied medium on the nickel state as a catalytic active component in the synthesized Ni/BCY15 cermets as an additional beneficial factor for the electrochemical activity of pSOFC anodes. For this purpose, a comparison was also performed between hydrazine-prepared Ni cermet and Ni cermet prepared by the commercial mechanical mixing procedure. The employed methodology involved combining the results from several methods, including X-ray powder diffraction, N_2_ physisorption measurements, scanning electron microscopy, electron paramagnetic resonance, X-ray photoelectron spectroscopy, and electrochemical impedance spectroscopy. To our knowledge, the analysis of the bare BCY15 ceramic matrix by electron paramagnetic resonance and X-ray photoelectron spectroscopy, as well as the detailed examination of the surface of Ni/BCY15 cermets by X-ray photoelectron spectroscopy, are novelties in the development of pSOFC anodes.

## 2. Materials and Methods

### 2.1. Materials

BaCe_0.85_Y_0.15_O_2.925_ (BCY15) powder (Marion Technology, Verniolle, France), used as the anode ceramic matrix, was fabricated by the auto-combustion process starting from metal nitrates and applying urea as a reducing agent. Sintering of the precursor at 1100–1150 °C in a carrier gas (helium or argon) for complete CO_2_ elimination ensured the production of single-phase powder with 48% porosity and a dominating particle size around 200 nm, and a minor degree of agglomeration. Before synthesis, the BCY15 powder was thermally pretreated at 1100 °C for 2 h.

Nickel chloride hexahydrate (NiCl_2_·6H_2_O), hydrazine monohydrate (99+% N_2_H_4_·H_2_O), sodium hydroxide (NaOH), and anhydrous sodium carbonate (Na_2_CO_3_), of analytical grade, all procured by Alfa Aesar (Ward Hill, MA, USA), were used.

### 2.2. Synthesis

Two Ni/BCY15 cermets of 50 g each were synthesized by wet-reduction with hydrazine using different media, such as deionized water and ethylene glycol (EG). These media were used for the preparation of the initial NiCl_2_·6H_2_O solution and as environment during the reduction reaction. The synthesis was performed according to the reported wet-reduction procedure [30,35]. A NiO to BCY15 volume ratio of 44.4:55.6 was determined with both Ni/BCY15 cermets to match the Ni metal content of 32 wt.%. A pre-set amount of N_2_H_4_·H_2_O solution was used to provide a N_2_H_4_ to Ni^0^ weight ratio of 6:1. This ratio was chosen based on studies of Huang et al. [37]. These authors have established that the optimal N_2_H_4_ to Ni^0^ molar ratio is 4, which ensured complete reduction of the Ni^2+^ ions to Ni^0^ in the solution free of Ni(OH)_2_. The reduction of hydrazine complex precursors proceeds via formation of Ni(OH)_2_, which is further decomposed by the equation: 2Ni(OH)_2_+N_2_H_4_→2Ni+N_2_+4H_2_O. The reason for using a higher amount of N_2_H_4_·H_2_O (N_2_H_4_:Ni^0^ = 6:1) was a possible reduction of Ce^4+^ from the BCY15 structure to Ce^3+^ via treatment with hydrazine, which would take place in a thin surface layer by analogy with the known ceria reduction by hydrazine [29,38]. The installation equipment consisted of a five-liter glass reactor with a stirrer and a steam jacket equipped with pH electrodes, a thermocouple, a reflux condenser, and peristaltic pumps. The mixture of nickel hydrazine complex and BCY15 powder was reduced to nickel black deposited on the BCY15 surface by addition of an appropriate amount of alkaline solution (a mixture of NaOH and Na_2_CO_3_) to keep a constant alkaline pH value upon heating to 95 °C. For the purpose of complete reduction, these reaction conditions were retained for 1 h under vigorous stirring. The suspension was washed with deionized water several times up to neutral pH and absence of Cl^−^ ions. The samples were dried in the air at 100 °C for 20 h. Both obtained samples were further denoted by an extension to indicate the applied medium, namely Ni/BCY15-W and Ni/BCY15-EG.

### 2.3. Catalyst Characterization

#### 2.3.1. Standard Characterization

N_2_ physisorption measurements were carried out on a Quantachrome Instruments NOVA 1200 e (Boynton Beach, FL, USA) apparatus by low-temperature (77.4 K) nitrogen adsorption after sample outgassing in a vacuum at 80 °C for 16 h. The nitrogen adsorption-desorption isotherms were analyzed through the linear part of the curves to evaluate the specific surface area calculated by means of the Brunauer–Emmett–Teller (BET) equation. Pore size distribution (PSD) was obtained by the Barrett–Joyner–Halenda method using the desorption branch of the isotherms.

X-ray powder diffraction (XRD) data were collected on a Bruker D8 Advance (Bruker-AXS, Karlsruhe, Germany) diffractometer employing CuKα radiation (λ = 0.15406 nm), operated at U = 40 kV and I = 40 mA. The crystalline phases were identified by means of International Centre for Diffraction Data (ICDD) powder diffraction files. The semi-quantitative analysis (as wt.%) was performed with the program Diffrac.Eva V4 (Diffrac.Eva Version 4, Bruker AXS GmbH, Karlsruhe, Germany) using the ICDD PDF-2 2021 database.

#### 2.3.2. Scanning Electron Microscopy (SEM)

The morphological studies were performed using a JEM 200 CX (Instruments, München, Germany) transmission scanning microscope equipped with ASID 3D.

#### 2.3.3. Electron Paramagnetic Resonance (EPR) Spectroscopy

Electron paramagnetic resonance measurements were performed at a temperature of 123 K using a JEOL JES-FA 100 EPR spectrometer (JEOL, Tokyo, Japan) operating at the X band (~9.8 GHz). The magnetic field was modulated at 100 kHz and the g values were determined from precise frequency and magnetic field values. The samples were placed in a quartz tube and fixed in the center of a standard TE011 cylindrical resonator. The EPR spectra were recorded under the following conditions: microwave power 5 mW, modulation amplitude 0.5 mT, sweep 500 mT, time constant 0.03 s, and sweep time 2 min.

#### 2.3.4. X-ray Photoelectron Spectroscopy

X-ray photoelectron spectroscopy (XPS) measurements were carried out on an AXIS Supra electron spectrometer (Kratos Analyical Ltd., Trafford Park, Stretford, United Kingdom) with a base vacuum in the analysis chamber of around 10^−8^ Pa. The samples were irradiated with Mg Kα photons with energy of 1253.6 eV. According to their kinetic energy, the photoemitted electrons were separated by a 180°-hemispherical analyzer with total instrumental resolution of ~1.0 eV (as measured by the FWHM (full width at half maximum) of the Ag3d_5/2_ line) at a pass energy of 20 eV. Due to the charging effect, a resolution of ~1.3 eV was measured on the isolated samples. Energy calibration was performed by normalizing the C1s line of adventitious adsorbed hydrocarbons to 285.0 eV. The analysis area was 300 × 700 μ^2^. The concentrations (as at.%) of the observed chemical elements were calculated by normalizing the areas of their most intense photoelectron peaks to their relative sensitivity factors using commercial spectrometer software (SpecsLab2 CasaXPS software, Casa Software Ltd, Bay House 5 Grosvenor Terrace Teignmouth, United Kingdom). The accuracy of the binding energy determination was within ±0.1 eV.

‘Bare’ anode samples were prepared using a standard ceramic technology by cold pressing (3 t/5 min) to obtain pressed tablets followed by sintering in air at 1200 °C for 5 h. Volumetric shrinkage of each tablet did not exceed 6%. The sintering procedure was used for real electrodes, leading to obtaining dense cermet tablets with definite porosity and grain size.

### 2.4. Electrochemical Measurements

The electrochemical impedance spectroscopy (EIS) measurements were performed on an IVIUM—CompactStat e10030 (Alvatek Ltd, Tetbury, England) in the temperature interval of 100–750 °C and frequency range of 1 MHz–0.01 Hz, with a density of 5 points/decade and amplitude of the AC signal of 1 mA in the reduction atmosphere. In situ redox cycling analysis was performed on two types of symmetrical half cells fabricated by tape casting of the cermet slurry on the BCY15 dense electrolyte support and sintered at 1350 °C for 4 h using the following compositions: (i) Ni/BCY15-Mech obtained by a ball-milled mixture of 30 vol.% BCY15 and 70 vol.% NiO (NOVAMET, HP green NiO-Type A) powders, and (ii) Ni/BCY15-EG powders prepared by wet-reduction with hydrazine in ethylene glycol.

The impedance measurements were preceded by a specially developed reduction procedure [39] performed at 800 °C in the reducing atmosphere, starting with the H_2_+N_2_ mixture and followed by reduction in pure hydrogen. This treatment transforms the NiO to Ni. Each sample was subjected to six redox cycles following the regime presented in Table 1.

## 3. Results and Discussion

### 3.1. X-ray Powder Diffraction

The basic analysis of the impact of the wet-chemical synthesis route on the structural characteristics of as-prepared Ni/BCY15 cermets was performed by XRD. The patterns of bare BCY15 and as-prepared Ni/BCY15 cermets were graphically presented in our previous study [30,35]. They exhibit the characteristic diffraction lines of single-phase isostructural BaCeO_3_ (ICDD-PDF file 00-022-0074). After Ni^0^ deposition on a BCY15 ceramic matrix, typical reflections of metallic Ni (ICDD-PDF file 00-004-0850) were observed and parasite BaCO_3_ phase (ICDD-PDF file 00-045-1471) was also registered, however, pattern intensities in this case of Ni/BCY15-EG were negligible [35] if compared with very well-organized and intense reflections of the BaCO_3_ phase observed in the aqueous environment [30]. However, at Ni/BCY15-W, only two phases were detected, namely metallic Ni and parasite BaCO_3_. There were no reflections for BaCeO_3_ structure (Table 2). As it was mentioned above, during synthesis, the deionized water solvent and medium in the reactor causes decomposition of the BCY15 perovskite structure and readily transforms part of the BaCe_0.85_Y_0.15_O_2.925_ material to barium oxide. Then, the latter reacts with carbon dioxide and water to yield barium carbonate [40]. Barium carbonate formation in ethylene glycol anhydrous synthesis is explained by partial decomposition of the BCY15 structure due to surface reduction of Ce^4+^ to Ce^3+^ by hydrazine, thus liberating some barium, which reacts with CO_2_ to BaCO_3_.

In this study, the semi-quantitative analysis was performed by XRD spectra to estimate the amount of parasite BaCO_3_ phase at both as-prepared Ni/BCY15 cermets listed in Table 2. It is evident that the amount of BaCO_3_ phase at Ni/BCY15-W is too much (60 wt.%), while the quantity is very low at Ni/BCY15-EG (4 wt.%). Indubitably, the application of anhydrous ethylene glycol medium leads only to a low extent of BCY15 structure decomposition. In addition to these comments, the great amount of BaCO_3_ is the reason for no registration of the main BaCeO_3_ perovskite structure.

It is also evident from the data in the Table 2 that Ni^0^ phase quantity at as-prepared Ni/BCY15-EG is higher (80 wt.%) than the theoretical Ni metal content of 32 wt.%. The explanation is the high mass absorption index of the nickel originated from its a large atomic number, thus leading to very good X-ray absorption. Another observation was that this Ni^0^ phase quantity is higher at Ni/BCY15-W (40 wt.%), due to the better covering of the BCY15 with metallic Ni particles and a lesser extent of decomposition of the BCY15 structure.

Our previous XRD examination [30,35] revealed that the sintering in air at 1350 °C, which is an obligatory stage in the technological cycle for anode tablets’ preparation and cannot be avoided, generates complete decomposition of parasitic BaCO_3_ phase at both Ni cermets. NiO and restored BaCeO_3_ perovskite phase as prevailing phases and formation of impurity phases were noticed, with a total amount of 6 wt.% obtained in sintered Ni/BCY15-W, as presented in Table 3. In contrast, XRD patterns of sintered BCY15/Ni-EG exhibited reflections of NiO, preserved BaCeO_3_ perovskite structure, and traces of 2% Y_2_BaNiO_5_ oxide.

The Ni/BCY15 anodes were processed in the reducing atmosphere before the impedance measurements to transform NiO to metallic Ni, noted in Table 3 as reduced anode tablets. The existence of BaNiO_2.36_ phase in the reduced Ni/BCY15-W anode indicated that the Ni ions are strongly included in this structure (2 wt.% amount), which is stable at exposure in the reducing atmosphere at 800 °C. The important finding is that after reduction treatment, only metallic Ni and the BCY15 structure existed in the Ni/BCY15-EG anode. The phase quantity of 92 wt.% Ni^0^ is due to the very intensive peaks of better-crystalized metallic nickel phase compared to as-prepared Ni/BCY15-EG cermet.

Data of the mean crystallite sizes (L_Ni_) of the formed metallic nickel in Table 2 clearly show a smaller size of the nickel in Ni/BCY15-EG, with 17%, compared to Ni/BCY15-W. This observation confirms ethylene glycol’s contribution as a reducing agent to decrease Ni^0^ size.

The XRD data show that no significant structural degradation was observed for the BCY15 ceramic matrix during wet-chemical synthesis of Ni/BCY15-EG as well as Ni/BCY15 anode tablets. Thus, the bulk structural analysis convincingly supports the advantage of the hydrazine wet approach via ethylene glycol assistance.

### 3.2. N_2_ Physisorption

The first common method for surface analysis of both the as-prepared cermets is N_2_ physisorption, which is known as a technique to study pore characteristics of solid materials. Pore size distribution (PSD) provides information about mesopores, which may facilitate the transport of reagent molecules to and from active sites, being an essential factor for catalyst design [41,42].

BCY15 support is a typical macroporous material, which is confirmed by the Type II isotherm characteristic of aggregated powders, such as clays, cements, etc., having a small H3-type hysteresis loop at the highest pressures [30]. Such an isotherm shape possessing a loop is referred to as a pseudo-Type II isotherm, adopted as Type II(b) [43,44]. Nickel deposition did not change the isotherm type of both Ni/BCY15 cermets, however, the hysteresis loops were increased in size relative to bare BCY15 [30,35]. The hysteresis loop type is also H3 due to mesopores being formed from non-rigid aggregates of plate-like particles [43,44]. The formation of clearly manifested hysteresis may be due to metallic Ni particles forming their own mesopore structure. The specific surface area increases more visibly with Ni/BCY15-EG (S_BET_ = 11 m^2^/g) in relation to that of BCY15 (Table 2).

**Figure 1:**
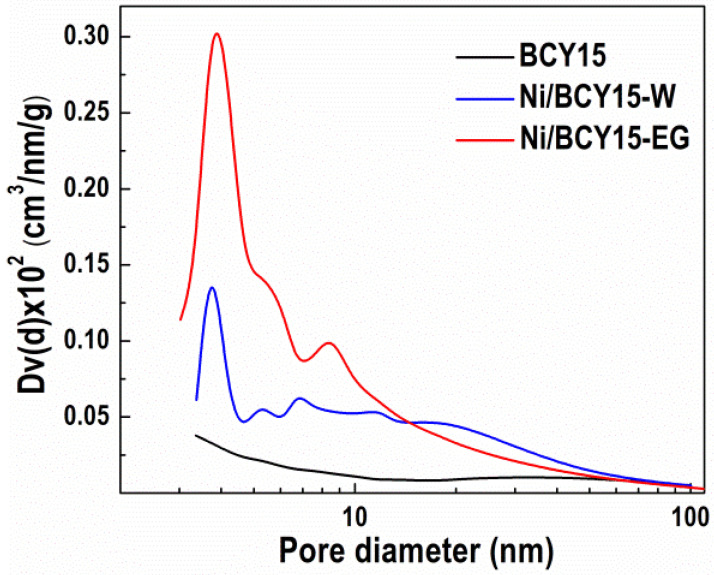
Pore size distribution of BCY15, Ni/BCY15-W, and Ni/BCY15-EG samples.

Pore size distribution of BCY15 and both nickel-containing samples is displayed in Figure 1. A uniform pore system of the BCY15 ceramic matrix is illustrated and the presence of some macropores above 50 nm indicates a macroporous material. The PSD curve of Ni/BCY15-W shows a polydisperse character in the 3–50 nm range. Compared with BCY support, new mesopores can be observed due to the formation of metallic Ni phase, with an average pore diameter (d_av_) of 11.5 nm (Table 2). In principle, this polydisperse character is the same after synthesis in EG medium, however, with intense maxima in the 3–14 nm range and less mesopores within 14–50 nm, with a lower d_av_ value of 9.1 nm (Table 2). Obviously, the formation of metallic nickel phase is accompanied by the creation of more mesopores. This observation could be explained by the presence of smaller Ni^0^ particles that expose smaller mesopores of 3–6 nm size, in comparison with Ni/BCY15-W PSD data. PSD intensity and shape within this range (Figure 1) indicate a relatively narrow distribution. The presence of larger amounts of pores in the Ni/BCY15-EG sample synthesized in ethylene glycol leads to a higher S_BET_ value (Table 2), as clearly seen in Figure 1.

Constant C in the BET equation is a fundamental parameter in the analysis of adsorption isotherms, which provides information about the magnitude of the adsorbent–adsorbate interaction force at the surface of the solids. The C constant values presented in Table 2 follow the range of 82–97. In general, Type II provides C values between 20 and 200 [45,46]. Changes in surface polarity may reflect the interaction of a quadrupole N_2_ molecule with a surface, leading to changes of the C parameter value. Based on the above-mentioned statements, the difference between BCY15 and Ni/BCY15-W values of C indicates an increase of the polarity due to the abundance of OH groups, which is a result of the interaction between the hydrophilic BCY15 and deionized water, leading to a partial decomposition of the initial BCY15 structure, as shown by XRD. A lower C value of Ni/BCY15-EG in comparison with Ni/BCY15-W presumes a decrease in surface polarity due to the effect of ethylene glycol medium and reflection to preserve the BCY15 structure. It is worth mentioning that the latter C value is also lower than that of the bare BCY15. The reduced surface polarity at Ni/BCY15-EG is consistent with more uniform coating of the BCY15 surface with metallic Ni particles and negligible decomposed BCY15 structure relative to Ni/BCY15-W. This fact favors the EG route synthesis. In addition, a larger amount of mesopores (higher S_BET_) in the Ni/BCY15-EG cermet facilitates the transport of reagent molecules to and from active sites.

### 3.3. Structural and Morphological Characterization

**Figure 2:**
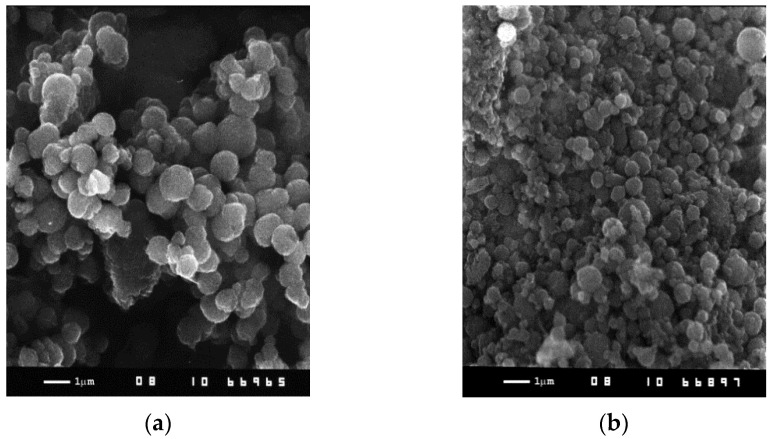
Micrographs of (**a**) Ni/BCY15-W and (**b**) Ni/BCY15-EG samples at 10,000× magnification.

The SEM technique was used to characterize the surface microstructure of deposited Ni^0^ on the surface of the BCY15 matrix for the same magnification. Image analysis shows that a different morphology was observed for as-prepared Ni/BCY15-W (Figure 2a) and Ni/BCY15-EG (Figure 2b) cermets.

The Ni^0^ particles in the Ni/BCY15-W cermet are characterized by different size (Figure 2a). The presence of agglomerated Ni particles forming bigger and smaller agglomerates during the wet-reduction synthesis in aqueous medium is clearly observed. Metallic nickel is unevenly distributed on the BCY15 surface. The possibility that part of the surface Ni^0^ particles will be covered with BaCO_3_ phase, formed after the partial destruction of the BCY15 perovskite structure, cannot be excluded either, as is shown by XRD (Table 2). XRD evidence further indicates (Table 2) that after partial destruction of the BCY15 perovskite structure, the surface Ni^0^ particles may be covered by BaCO_3_ phase to a certain extent.

A SEM image of the Ni/BCY15-EG cermet surface reveals that the microstructure seems homogeneous, exposing spherical particles of similar size and no sign of agglomeration (Figure 2b). Undoubtedly, ethylene glycol anhydrous synthesis provides uniformly distributed Ni^0^ particles of smaller particle size.

It is well-known that high metal loadings affect metal dispersion on the support. However, the use of ethylene glycol as a protective solvent saves not only the BCY15 structure but also, owing to its properties as a reducing and dispersive agent, leads to a finely divided metal Ni phase of smaller particle sizes.

### 3.4. Electron Paramagnetic Resonance

EPR spectroscopy characterized by high selectivity and sensitivity (10^−11^–10^−12^ mol/L) was used to gain further information about the bulk nature of both the bare BCY15 matrix and active metallic Ni phase to find detailed explanations for differences in the electrochemical activity of Ni/BCY15-W and Ni/BCY15-EG anodes. So far, EPR analysis of the yttrium-doped barium cerate (BaCe_0.85_Y_0.15_O_2.925_; BCY15) structure is still lacking in the current literature.

**Figure 3:**
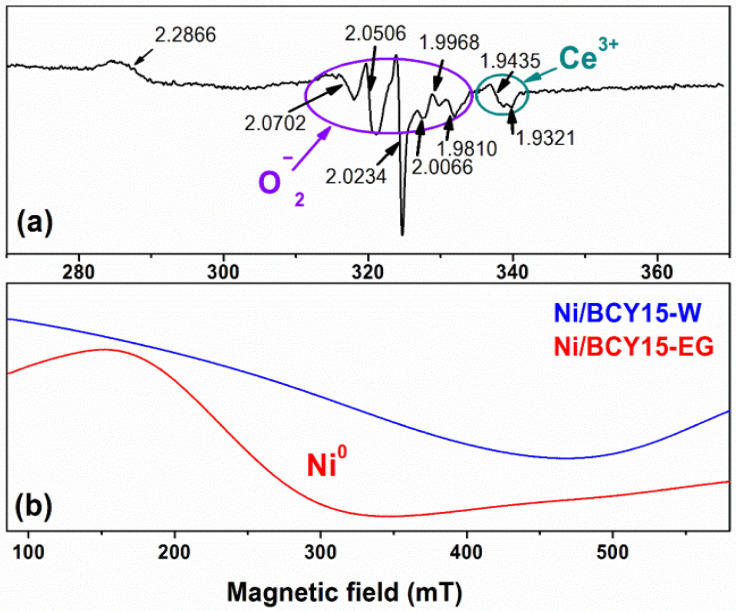
EPR spectra recorded at 123 K: (**a**) bare BCY15 and (**b**) Ni/BCY15-W and Ni/BCY15-EG cermets.

Several signals have been registered in the EPR spectrum of bare BCY15 (Figure 3a). One of them, with g_⊥_ = 1.9436 and g_||_ = 1.9321, is associated with the presence of Ce^3+^ sites, narrow lines marked with a circle in Figure 3 due to O^2−^ species on the cerium surface (Ce^4+^ − O^2−^), and a signal with g = 2.2866, which could be ascribed to Ce^3+^ in a distortion polyhedron of defect associations {Ce_Ce_’VÖ} [47]. The g-factor values have small deviations compared to the typical EPR signals of Ce^3+^ ion and oxygen vacancies reported in the literature [38,48,49], which can be attributed to the incorporated Y^3+^ ions which partially replaced Ce^4+^ host cations into the BCY15 structure by creation of oxygen anion vacancies and Ce^3+^ sites, in agreement with a study of Y-doped CeO_2_ [50].

After metallic Ni deposition, broad lines were only detected in the EPR spectra of as-prepared Ni/BCY15-W and Ni/BCY15-EG cermets (Figure 3b). It is good to mention that unlike a simple EPR signal of ceria reduced in H_2_ at 500 °C, hydrazine-treated ceria displays a complicated EPR spectrum with signals at g-values of 1.979, 1.998, and 2.013 [38]. However, the high amount of nickel (32 wt.% metallic Ni) makes it impossible to record signals corresponding to paramagnetic Ni^0^ and Ni^2+^, as well as Ce^3+^ ions. However, because of the broad EPR spectrum of freshly prepared Ni/BCY15 cermets, according to literature data, the Ni^2+^ ions may originate from probable superficial reoxidation of the metallic Ni particles, in part after the hydrazine reduction during subsequent washing and drying stages [29]. It has been established that the interaction between metallic nickel phase and ceria support in hydrazine Ni/CeO_2_ catalysts highly preserves the nickel from partial oxidation in air [29]. The EPR data show that the Ni/BCY15-EG spectrum is better resolved. This narrowing of the signal supposes that the interaction between hydrazine-originating metallic Ni and cerium from the BCY15 matrix in the anhydrous ethylene glycol environment is stronger than in aqueous medium.

Apparently, the magnitude of the nickel–cerium interaction would be an additional factor for better electrochemical performance of the Ni/BCY15-EG anode, combined with smaller and more uniform size of better-distributed metallic Ni particles.

### 3.5. X-ray Photoelectron Spectroscopy

Surface structure properties of Ni/BCY15-W and Ni/BCY15-EG cermets are an important factor that controls the performance of these materials as anodes. X-ray photoelectron spectroscopy is a powerful and sensitive probe for surface examination and ensures reliable identification of the surface composition, oxidation states, and the relative dispersion of the components. Determination of the nickel chemical state on the BCY15 surface in as-prepared Ni/BCY15-W and Ni/BCY15-EG cermets is important because nickel is the key to adsorption, dissociation, and oxidation of the hydrogen, and an electronic conductor that provides the electronic conductivity of the anode.

**Figure 4:**
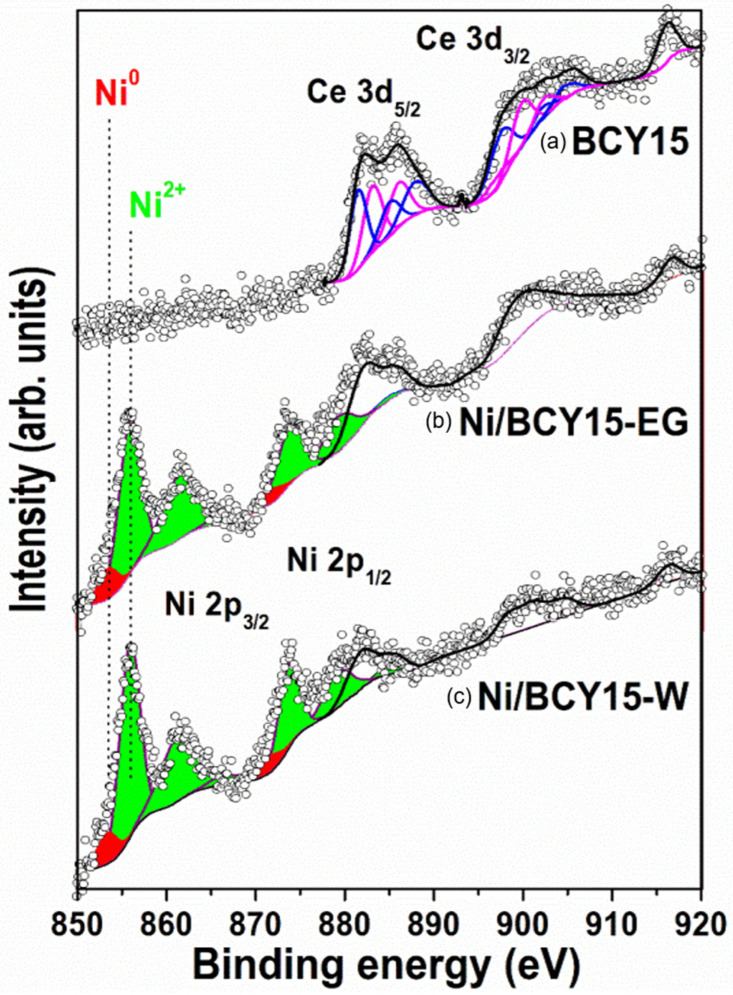
Ni2p and Ce3d photoelectron regions of the studied samples: (**a**) BCY15, (**b**) Ni/BCY15-EG, and (**c**) Ni/BCY15-W. Ni^0^ and Ni^2+^ species are colored in red and green, respectively. Black contour lines outline the total Ce3d signals from the studied samples. Contributions of the Ce^3+^ and Ce^4+^ oxidation states in spectrum (**a**) are marked in purple and blue, respectively.

The main Ni2p_3/2_ peaks with asymmetry toward a lower binging energy (BE), accompanied by shake-up satellite lines, were registered at 856.0 eV in the spectra of both Ni/BCY cermets (Figure 4, Table 4). The position of the peaks clearly indicates the presence of Ni^2+^ ions on the surface, which can be attributed to Ni(OH)_2_ (855.3–856.6 eV) [51,52,53]. It should be noted that the XRD data show only the presence of Ni^0^, while the Ni^2+^ oxidation state originates from surface oxidation of metallic Ni after the hydrazine reduction during subsequent water washing and air-drying stages, as already mentioned [29].

Curve fitting of the Ni2p level shows that the low-energy peak registered at 853.4 eV (Table 4) is present in the spectra of both samples (Figure 4). According to literature data, the Ni 2p_3/2_ spectrum of pure nickel metal is registered in the range of 852.7–853 eV [51,52,53,54]. Following the above results, the BE value corresponds to the presence of Ni^0^ on the surface. A small shift to higher binding energies is a result of the influence of the nearest neighbors around the metallic nickel particles [53]. Thus, this shift can be attributed to the established interaction of the metallic Ni particles with Ce^3+^ sites [50,55]. The Ce^3+^ sites are additionally formed in the structure of the BCY15 support by thin surface layer reduction of Ce^4+^ during hydrazine wet-reduction synthesis of both as-prepared Ni/BCY15-W and Ni/BCY15-EG cermets, similar to the studies of ceria reduction by the hydrazine reduction technique [29,38]. Contributions of metallic Ni and the Ni^2+^/Ni^0^ ratio are presented in Table 4. The data reveal that a larger amount of Ni^0^ is present on the surface of the Ni cermet synthesized in ethylene glycol anhydrous medium (Ni/BCY15-EG).

The claim stated above is based not only on the EPR data, but also on determining the Ce chemical state through analysis of the Ce3d photoelectron region. For this purpose, the surface analysis of bare BCY15 was performed as a reference point (Figure 4a). So far, no Ce3d spectrum of BCY15 has been reported in the literature.

Reference data using appropriate standards for the various chemical states of Ce [56,57] indicate that the BCY15 spectrum can be fitted with the characteristic spectra of the Ce^4+^ and Ce^3+^ oxidation states. Their peak contributions are marked in blue and purple, respectively. Deconvoluted XPS spectra contain many satellite peaks, which can be separated in two multiplet groups with spin-orbital splitting of 18.4 and 18.7 eV for Ce^4+^ and Ce^3+^, respectively. According to the literature [56], the origin of Ce^4+^ and Ce^3+^ is due to the final 4f occupied state connected with O2p-Ce4f charge transfer during photoemission. The highest binding energy peak at 916.1 eV is characteristic of the Ce^4+^ oxidation state without any contribution by Ce^3+^ ions. The detection of this peak in the spectrum unambiguously indicates the presence of the Ce^4+^ oxidation state in the Ce-containing complex compounds. Wang and Meng [57] and Shyu et al. [58] have proposed a methodology for determining the relative Ce^4+^ concentration compared with that of Ce^3+^ by using the relative area of the highest binding-energy satellite peak to the total Ce3d area. Results of the studied samples are presented in Table 5. As can be seen, the relative concentrations of Ce^4+^ and Ce^3+^ in BCY15 and Ni/BCY15-EG are similar, 62% and 65% respectively, and increase significantly to 84% for Ni/BCY15-W. Therefore, analysis of the Ce3d spectra by deconvolution shows coexistence of the Ce^4+^ and Ce^3+^ oxidation states, in agreement with literature data [59,60,61,62,63]. The presence of Ce^3+^ sites in BCY15 was also found by EPR, caused by Y^3+^ ion-doping in the matrix.

The position of the Ce^4+^ satellite registered at 916.1 eV in BCY15 (Figure 4, Table 4) is shifted toward higher BEs after metallic Ni deposition at 916.3 and 916.7 eV for Ni/BCY15-W and Ni/BCY15-EG, respectively. The shift of the Ce3d line is larger than ±0.1 eV experimental accuracy and it can be explained by different electron densities (different bond lengths) around the cerium atoms after introduction of metallic Ni particles in the BCY15 matrix. Bearing in mind the above-mentioned results of Ni^0^ and Ni^2+^ BEs, namely that metallic Ni is situated at a BE of 853.4 eV (Table 4) and the Ni^2+^ oxidation state arose from partial surface reoxidation of metallic Ni particles to Ni(OH)_2_, the observed Ce^4+^ satellite shift could be indicative of the interaction strength between metallic Ni and Ce^3+^ species. The satellite position is higher by 0.4 eV in the Ni/BCY15-EG spectrum compared with that of Ni/BCY15-W, and by 0.6 eV relative to BCY15. It is known that the chemical shift of the XPS peak (ΔBE) serves to evaluate the degree of interaction between the individual components in the studied system. Due to the higher BE value of 916.7 eV of the Ni/BCY15-EG cermet, the kinetic energy is lower (E_kinetic_ = hν − E_binding_). This affords a possibility to claim that upon reduction, the formed Ni^0^–Ce^3+^ bond is stronger after synthesis using ethylene glycol as a medium and reducing agent. The observation is also in agreement with better-resolved EPR spectra (Figure 3).

Considering the data in Table 5, it follows that the relative concentration of the Ce^3+^ ions with respect to all Ce ions is 38% in the surface layers of the BCY15 sample. It decreases slightly to 35% with Ni/BCY15-EG and reaches 16% in the BCY15/Ni-W sample, showing that the Ce^3+^ sites are twice as many on the surface of BCY15/Ni-EG cermet after ethylene glycol anhydrous application, relative to aqueous medium in N_2_H_4_-reduction synthesis. Since Ce^3+^ ions in hydrazine-treated ceria are stable only in the presence of N_2_H_4_ and pure CeO_2_ is restored upon drying of the reduced powder in air, the data on the relative Ce^3+^ concentration in both Ni/BCY15 samples points to a better-stabilized Ce^3+^ oxidation state on the BCY15/Ni-EG cermet surface due to the bond with metallic Ni.

**Figure 5:**
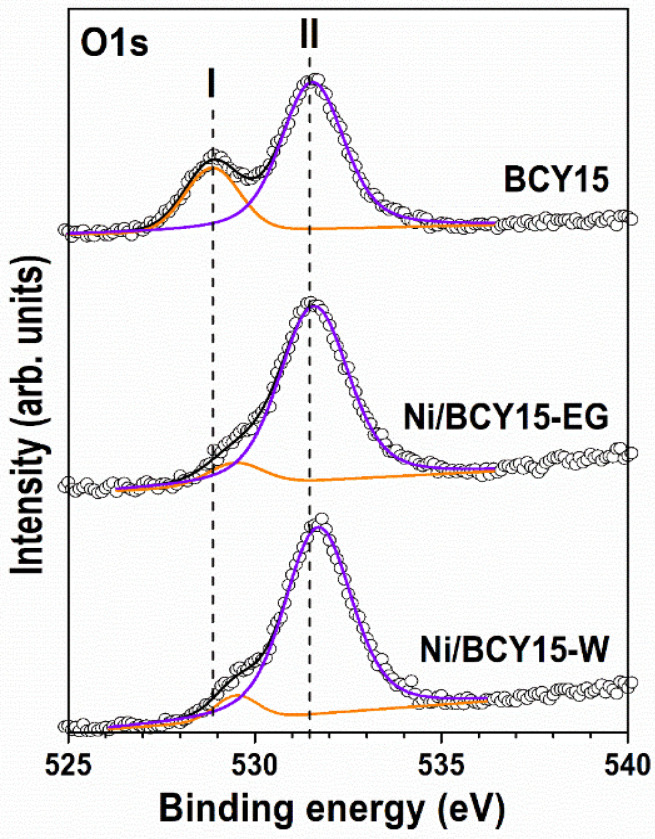
O1s level of the BCY15 surface in as-prepared Ni/BCY15-W and Ni/BCY15-EG cermets.

The O1s spectrum consists of two peaks, namely a less intensive low-energy peak at 528.8 eV and a most intense higher energy peak at 531.5 eV, henceforth noted for convenience as peak I and peak II (Figure 5, Table 4). References for analysis of the O1s level of BCY15 are lacking in the current literature.

It is known that a low binding energy peak at 528.7–529.2 eV in the O1s spectrum of pure CeO_2_ originates from lattice oxygen atoms (O^2−^), and a high binding energy peak at 531.5–532.0 eV is formed from hydroxyl groups [59,60,63,64]. On this basis, peak I can be attributed to oxygen atoms in the lattice of BCY15 (BaCe_0.85_Y_0.15_O_2.925_), whereas peak II can be recognized as due to physisorbed hydroxyl groups coming from atmospheric moisture uptake as a result of yttrium-doped barium cerate matrix affinity to H_2_O [40].

A considerably broader main O1s peak, being asymmetrical on the low binding energy side, characterizes the same spectra of as-prepared Ni/BCY15-W and Ni/BCY15-EG cermets. It is obvious that the low-energy peak I with BCY15 is transformed into a shoulder (Figure 5), as better outlined for Ni/BCY15-W. Peak I in the Ni/BCY15-W and Ni/BCY15-EG spectra can be associated with O^2−^ in Ce–O bonding. Compared with bare BCY15, there is a noticeable decrease in intensity of these peaks, which could be related to Ce^4+^ reduction. The same observation has been reported for the Ni/CeO_2_ system [65]. Besides, as deconvolution results show, the peaks are shifted to higher BE at 529.4 eV for both Ni/BCY15 cermets (Figure 5, Table 4). These observations are because of two simultaneous processes: surface reduction of Ce(IV) to Ce(III) by loss of lattice oxygen and Ni interaction with cerium during the hydrazine reduction process. The aforementioned statement is supported by literature data on binding energies of oxygen ions in oxygen-deficient regions caused by oxygen vacancies (O_x_^−^) in a matrix of metal oxides registered at 529.9–531.1 eV [65,66,67,68,69]. The O1s peak for the reduced state of cerium, oxygen in the Ce_2_O_3_ lattice, is also registered at higher BE values, namely 529.8–530 eV [60,63]. Evidently, a lower peak intensity in combination with peak position in the spectrum of Ni/BCY15-EG cermet can be associated with a deeper reduction of cerium, and it is a further indication for a stronger Ni^0^–Ce^3+^ interaction in ethylene glycol-assisted synthesis.

The spectra exhibit a second symmetrical high binding energy peak. This peak, denoted as peak II, is centered at the same BE of 531.5 eV for Ni/BCY15-EG and shifted to 531.7 eV for Ni/BCY15-W, i.e., more than an instrument accuracy of ±0.1 eV. Both peak II values are more intense related to the BCY15 spectrum. They originate from two types of oxygen: OH groups from Ni(OH)_2_ and physisorbed humidity, and oxygen in CO_3_ groups due to partial decomposition of the BCY structure to BaCO_3_, as evidenced through the amount of BaCO_3_ phase at Ni/BCY15-W of 60 wt.% calculated by the semi-quantitative XRD analysis (Table 2). The O1s of pure BaCO_3_ has been registered at 531.1 eV [70]. Therefore, it is not possible to determine the contribution of each type of oxygen to the position of peak II.

Attention was also paid to another important factor—relative dispersion of the components on the surface. An estimation of nickel atoms’ dispersion was performed by calculation of the Ni/(Ba+Ce+Y) ratio and is summarized in Table 5. Thus, Ni dispersion on the BCY15 surface is higher for the Ni/BCY15-EG cermet, as already shown by PSD (Figure 1), while the SEM image (Figure 2b) indicates that the BCY15 surface is uniformly covered by deposited smaller metallic Ni particles.

Another intention was to evaluate the BCY15 surface after the synthesis of both Ni cermets. A Ba/(Ce+Y) ratio was calculated (Table 5) to consider partial decomposition of the BCY matrix during synthesis and liberation of barium. A value of 1.52 for Ni/BCY15-W relative to 0.63 for bare BCY15 proved that there was more of a presence of barium on the surface due to the formation of more BaCO_3_ using aqueous medium. However, Ni/BCY15-EG manifested a lower value (0.35) by comparison with BCY15, which undoubtedly confirms that the BCY15 surface is more uniformly covered with nickel atoms, resulting in shielding of the signal from the other chemical elements, thus being indirect proof for better deposition.

In summary, the surface science study showed that nickel dispersion is increased by using the ethylene glycol environment and that metallic Ni is better stabilized over a partially reduced cerium surface due to a stronger interaction. As a result, further evaluation proceeded with the BCY15/Ni-EG anode.

### 3.6. Electrochemical Performance

The electrical properties of the anodes prepared with EG were evaluated using comparative electrochemical impedance analysis in respect of symmetrical half-cells prepared from Ni/BCY-Mech. Each half-cell was subjected to six redox cycles following the standardized procedure presented in Table 1. Samples of Ni/BCY15-W were excluded from the tests since their composition does not correspond to the required one due to the strong decomposition of the BCY15 structure in accordance with XRD data.

As already mentioned, during sintering in air, which is an obligatory stage in the technological cycle that cannot be avoided, the initial metallic Ni used for synthesis of Ni/BCY15-EG oxidizes partially or fully to NiO. In principle, the process is associated with volume expansion and should cause stresses on the electrolyte matrix and the appearance of cracks or delamination [71]. However, the application of the EG technological approach ensures production of well-dispersed nanosized metallic nickel particles on the BCY15 matrix and provides a strong metal–partially reduced cerium interaction through Ce^3+^ sites, determining the electrochemical activity of nickel. Both factors suppose improved electrochemical characteristics and increased stability, which is a prerequisite for decreased anode degradation under operating conditions. In addition, the nanometric Ni phase would ensure easier spills during oxidation, and instead of forming cracks, this will increase the voids, thus improving the tolerance with respect to Ni agglomeration and redox cycling [28].

In principle, redox cycling can be considered an accelerated stress test regarding Ni cermet degradation, since it leads to similar degradation mechanisms, namely Ni coarsening and Ni depletion, which decrease the triple-phase boundary points and thus the catalytic activity and conductivity.

**Figure 6:**
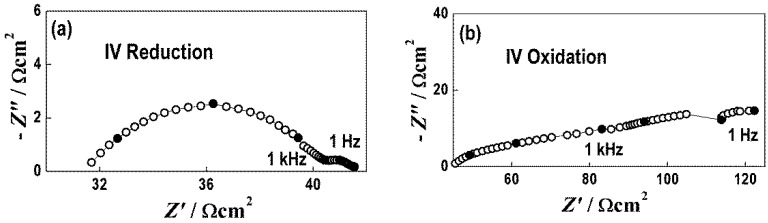
Impedance diagrams of reduced (**a**) and oxidized (**b**) states (4th cycle) of symmetrical half-cells with the Ni/BCY-Mech anode.

**Figure 7:**
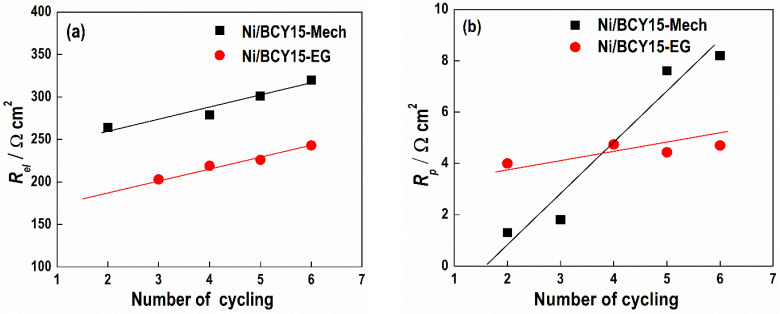
Dependences of electrolyte resistance (**a**) and anode polarization resistance (**b**) on the number of cycles for Ni/BCY15-EG (●) and Ni/BCY15-Mech samples (■).

Figure 6 displays typical impedance diagrams of reduced and oxidized states, which are used for evaluation of the electrolyte resistance and the polarization resistance of the Ni/BCY15-Mech anode, presented as area-specific resistance (ASR). A summary of the results from the impedance measurements is provided in the form of Arrhenius plots (Figure 7).

The results of the electrolyte resistance show that during reoxidation, it increases in a similar way for both samples, Ni/BCY15-EG and Ni/BCY15-Mech (Figure 7a). The different values of the electrolyte resistance, 88% and 90% for Mech and EG samples respectively, are probably due to the different porosity of the electrolytes.

Bearing in mind the electrode polarization resistance, however, a drastic increase with the Ni/BCY15-Mech sample was observed. Although the initial resistance was lower for the pristine sample, on the 6th cycle, it was already two times higher than that of the Ni/BCY15-EG sample, i.e., a very fast degradation rate was observed (Figure 7b). This result confirms the higher stability of the microstructure obtained by the EG method in relation to Ni coarsening and migration. The smaller size of the Ni particles and thus bigger pore sizes also ensure higher mechanical stability.

Thus, the observed electrochemical behavior of Ni/BCY15-EG anodes confirms that the method of wet-reduction by the hydrazine reducing agent guarantees better dispersion of the Ni metal particles in the BCY15 matrix, minimizing the microstructural changes during its reoxidation, and accordingly, strongly influences the degradation processes and whole cell performance.

## 4. Discussion and Summary

Analysis of the nickel state in the bulk and on the surface of the BCY15 matrix provided an opportunity to discover the origin of the differences between two cermets (Ni/BCY15-W and Ni/BCY15-EG) prepared via aqueous and ethylene glycol environments.

XRD analysis showed that the hydrazine wet-reduction methodology ensured complete reduction of the formed Ni^2+^ hydrazine complex ([Ni(N_2_H_4_)_x_]Cl_y_) to Ni^0^ particles. An advantage of this route is the easy preparation of fine nickel powders at a low reaction temperature (80–95 °C) and a simple procedure compared with the classical synthesis method, which involves a solid-state reaction between NiO powder and BCY electrolytes at high temperatures of 1100–1400 °C, followed by hydrogen reduction at a temperature of 700–800 °C.

Our findings showed that the hydrazine wet-reduction approach to synthesize Ni/BCY15-EG cermet in ethylene glycol medium provides a metallic Ni phase in the pSOFC anode that is more stable during reoxidation compared to Ni/BCY15-Mech cermet prepared by the commercial mechanical mixing procedure. This fact clearly outlines another advantage of this method for metallic Ni incorporation into the electrolyte matrix. Ethylene glycol, used not only as anhydrous medium but also as an additional reducing agent, provided nano-scaled Ni^0^ particles of narrow size distribution, a higher specific surface, and improved dispersion on Ni/BCY15-EG cermet (XRD, N_2_ sorption, SEM, XPS). These features facilitated fuel molecules’ access to the nickel sites. As a result, more nickel in the Ni/BCY15-EG anode responds faster to the hydrogen, and thus the percolation process is more rapidly achieved, and full connectivity among the metallic Ni particles during creation of the conductive network in the electrode is attained [30,35].

Another determining factor for better electrochemical performance of the Ni/BCY15-EG anode if compared with Ni/BCY15-W (XPS, EPR) is the interaction magnitude between nickel and cerium in the as-prepared cermet. The suggestion is that the nickel–cerium interaction has arisen during decomposition of the Ni^2+^ hydrazine complex, followed by transformation of the Ni^2+^ ions into metallic Ni. It is useful to note that a mutual influence of nickel and ceria exists during the N_2_H_4_-reduction process [29]. A stronger interaction in as-prepared Ni/BCY15-EG definitely takes place in the bulk BCY15 structure (EPR), not only on the surface (XPS). It is assumed that Ni^0^ stabilization occurs through bonding to Ce^3+^ sites formed by lattice oxygen transfer. The interaction affinity is kept after reoxidation during electrode preparation, and it is the reason for a completely formed conductive Ni^0^ network characterized by low resistance after 24 h exposure of the cell to ambient conditions. Stabilization of the metallic nickel state on the surface determines its resistance to oxidation and offers an increased number of interacting metal atoms, leading to better electronic conductivity. Consequently, this is a precondition for better durability of the Ni/BCY15-EG anode.

Our finding is in agreement with a fundamental study of the Ni/CeO_2_ system [62]. The authors have detected uniform distribution of Ni particles over ceria surface, and this has been explained as a result of surface defects (oxygen vacancies) being the nucleation sites for nickel. It is stated that the Ce^3+^ sites do not only exist in the regions near the metal, i.e., at the metal–ceria interfaces [50]. In a theoretical study with density functional theory (DFT) calculations applied for estimation of Ni deposition on stoichiometric (CeO_2_) and reduced (Ce_2_O_3_) cerium oxide surfaces, Liu et al. have claimed that the nickel oxidation state is 2+ on CeO_2_, and it remains metallic over reduced Ce_2_O_3_ [55]. The presented model surface shows that Ni^0^ interacts with Ce^3+^ sites. In addition, Lustemberg et al. have shown a change occurring under reduction conditions: Ni^2+^−CeO_2_→Ni^0^−CeO_2−x_. Upon increasing Ce^4+^ reduction by oxygen removal, the oxidation state of nickel was transformed from +2 to 0 [72]. The authors also revealed that the most stable Ni^0^ particles are located on O-bridge sites, whereas O-top and O-hollow sites are less stable. Hence, Ni^0^ is stabilized by bonding to Ce^3+^ sites that arise during hydrazine reduction.

By the same analogy, uniform distribution of nickel nanoparticles in Ni/BCY15-EG cermet is suggested by an increase of the oxygen vacancies on the BCY15 surface and the accompanying stronger interaction between metallic Ni and Ce^3+^ sites in the BCY15 matrix due to deeper reduction by ethylene glycol. Furthermore, the interaction between cerium and Ni could also change the electronic properties of nickel [62], thus affecting its electrochemical reactivity.

The application of the wet-chemical synthesis route in an anhydrous ethylene glycol environment provided not only anode ceramic matrix structure preservation, but also a new microstructure of the Ni/BCY15 pSOFC anode more tolerant to redox cycling, i.e., the microstructure is more stable to changes causing degradation. The advantage of the hydrazine wet-reduction methodology for improvement of the electrochemical behavior of the Ni/BCY15 pSOFC anode is indubitable.

## Figures and Tables

**Table 1:** Redox cycling regime.

Oxidation			Reduction		
Duration	N_2_	Air	Duration	N_2_	H_2_
(Min)	(mL/Min/cm^2^)	(mL/Min/cm^2^)	(Min)	(mL/Min/cm^2^)	(mL/Min/cm^2^)
2	3.97	3.97	6	3.97	3.97
6	3.97	0	10	22.28	0
			5	3.97	0

**Table 2:** XRD and N_2_ physisorption parameters of as-prepared Ni/BCY15 cermets.

Sample	Phase	Phase Quantity, wt.%	L_Ni(111)_ nm	S_BET_ m^2^/g	d_av_ nm	Constant C
BCY15	BaCeO_3_	−		3	14	90
Ni/BCY15-W	Ni^0^	40	15.7	8	11.5	97
	−	−				
	BaCO_3_	60				
Ni/BCY15-EG	Ni^0^	80	13.0	11	9.1	82
	BaCeO_3_	16				
	BaCO_3_	4				

**Table 3:** XRD parameters of sintered and reduced Ni/BCY15 anode tablets.

Stage	Ni/BCY15-W		Ni/BCY15-EG	
	Phase	Phase Quantity, wt.%	Phase	Phase Quantity, wt.%
Sintered anode tablets	NiO	40	NiO	68
	BaCeO_3_	54	BaCeO_3_	30
	BaNiO_2.36_	3	Y_2_BaNiO_5_	2
	Y_0.10_Ce_0.90_O_1.95_	3		
Reduced anode tablets	Ni^0^	80	Ni^0^	92
	BaCeO_3_	17	BaCeO_3_	8
	BaNiO_2.36_	2		
	Y_0.10_Ce_0.90_O_1.95_	1		

**Table 4:** Binding energies of the peaks of the BCY15 surface in as-prepared Ni/BCY15-W and Ni/BCY15-EG cermets, referred to as Cls, with BE = 285.0 eV.

Sample	Binding Energy (eV)				
	Ni2p		Ce3d	O1s	
	Ni^0^	Ni^2+^	Ce^4+^ Satellite	I	II
BCY15	−	−	916.1	528.8	531.5
Ni/BCY15-W	853.4	856.0	916.3	529.4	531.7
Ni/BCY15-EG	853.4	856.0	916.7	529.4	531.5

**Table 5:** XPS data on the BCY15 surface of as-prepared Ni/BCY15-W and Ni/BCY15-EG cermets.

Sample	Ni^2+^/Ni^0^ Ratio	Contribution, %		Ni/(Ba+Ce+Y)	Ba/(Ce+Y)
		Ni^0^	Ce^4+^		
BCY15	−	−	62	−	0.63
Ni/BCY15-W	10.4	8.8	84	1.82	1.52
Ni/BCY15-EG	9.9	9.2	65	1.94	0.35

## Data Availability

The data presented in this study are available in the article.
